# 4-{(*E*)-*N*′-[2-(8-Quinolyloxy)acetyl]hydrazonomethyl}benzoic acid methanol solvate

**DOI:** 10.1107/S1600536809021576

**Published:** 2009-06-17

**Authors:** Chun-Yan Ren

**Affiliations:** a College of Chemistry and Pharmacy, Qingdao Agricultural University, Shandong 266109, People’s Republic of China

## Abstract

In the title compound, C_19_H_15_N_3_O_4_·CH_4_O, the mean planes of the benzene ring and the quinoline system make a dihedral angle of 6.7 (2)°. The acetohydrazide host mol­ecules are connected *via* inter­molecular O—H⋯O hydrogen bonds into two-dimensional zigzag sheets extending in the *ab* plane. The methanol solvent mol­ecule is linked to the host mol­ecule *via* inter­molecular N—H⋯O and O—H⋯N hydrogen bonds.

## Related literature

For the coordination chemistry of 8-hydroxy­quinoline and its derivatives, see: Chen & Shi (1998 ▶). For a related structure, see: Wen *et al.* (2005 ▶). For bond-length data, see: Allen *et al.* (1987 ▶).
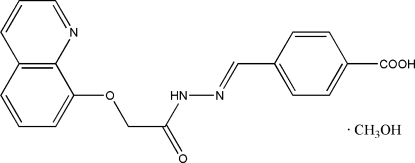


## Experimental

### 

#### Crystal data

C_19_H_15_N_3_O_4_·CH_4_O
*M* *_r_* = 381.38
Monoclinic, 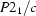

*a* = 10.1166 (18) Å
*b* = 11.095 (2) Å
*c* = 18.510 (3) Å
β = 115.896 (7)°
*V* = 1869.0 (6) Å^3^
*Z* = 4
Mo *K*α radiation
μ = 0.10 mm^−1^
*T* = 295 K
0.22 × 0.19 × 0.18 mm

#### Data collection

Bruker SMART CCD area-detector diffractometer
Absorption correction: multi-scan (*SADABS*; Sheldrick, 1996 ▶) *T*_min_ = 0.979, *T*_max_ = 0.982
9663 measured reflections
3302 independent reflections
1666 reflections with *I* > 2σ(*I*)
*R*_int_ = 0.046

#### Refinement

*R*[*F* ^2^ > 2σ(*F* ^2^)] = 0.054
*wR*(*F* ^2^) = 0.161
*S* = 1.02
3302 reflections
255 parameters
H-atom parameters constrained
Δρ_max_ = 0.20 e Å^−3^
Δρ_min_ = −0.20 e Å^−3^

### 

Data collection: *SMART* (Siemens, 1996 ▶); cell refinement: *SAINT* (Siemens, 1996 ▶); data reduction: *SAINT*; program(s) used to solve structure: *SHELXS97* (Sheldrick, 2008 ▶); program(s) used to refine structure: *SHELXL97* (Sheldrick, 2008 ▶); molecular graphics: *SHELXTL* (Sheldrick, 2008 ▶); software used to prepare material for publication: *SHELXTL*.

## Supplementary Material

Crystal structure: contains datablocks global, I. DOI: 10.1107/S1600536809021576/cs2119sup1.cif

Structure factors: contains datablocks I. DOI: 10.1107/S1600536809021576/cs2119Isup2.hkl

Additional supplementary materials:  crystallographic information; 3D view; checkCIF report

## Figures and Tables

**Table 1:** Hydrogen-bond geometry (Å, °)

*D*—H⋯*A*	*D*—H	H⋯*A*	*D*⋯*A*	*D*—H⋯*A*
O3—H3⋯O2^i^	0.82	1.88	2.695 (3)	171
O5—H5⋯N1	0.82	1.95	2.765 (3)	171
N2—H2⋯O5	0.86	2.01	2.838 (4)	162

Symmetry code: (i) 
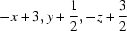
.

**Fig. 1.**
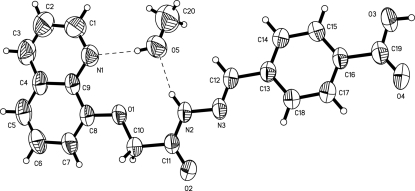
The asymmetric unit structure of the title compound, with displacement ellipsoids drawn at the 50% probability level. The dashed lines indicate hydrogen bonds.

**Fig. 2.**
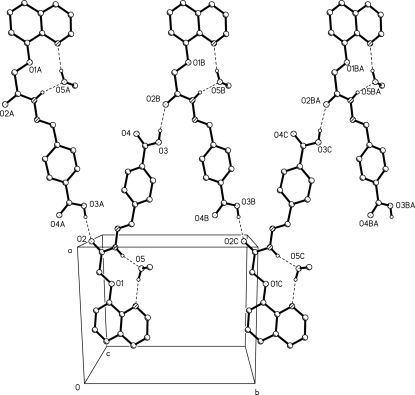
The H-bonding sheet from the crystal structure supported via hydrogen bridges (dashed lines), with only relevant H atoms shown for clarity.

## supplementary crystallographic information

### Comment

8-Hydroxyquinoline and its derivatives constitute well known ligands in coordination chemistry (Chen & Shi, 1998). As part of our on going search for good extractants of metal ions or a biologically active material, the title compound was obtained in the reaction of quinolin-8-yloxyacetic acid hydrazide and 4-formylbenzoic acid. In the crystal structure of all bond lengths and angles are normal (Allen *et al.*, 1987), and are comparable to those in the related compound *N'*-(2-Fluorobenzylidene)-2-(quinolin-8-yloxy)-acetohydrazide methanol solvate (Wen *et al.*, 2005). The mean planes of the benzene ring and the quinoline ring make a dihedral angle of 6.7 (2)°. In the crystal structure, the methanol molecule is linked to the C_19_H_15_N_3_O_4_ host molecule *via* intermolecular N—H···O and O—H···N hydrogen bonds (Fig. 1). Intermolecular O—H···O hydrogen bonds (Table 1) fuse the molecules into two-dimensional zig-zag sheets along the *a*-b** plane (Fig. 2).

### Experimental

2-(quinolin-8-yloxy)acetohydrazide (2.18 g, 10 mmol), 4-formylbenzoic acid (1.50 g, 10 mmol), ethanol (40 ml) and some drops of acetic acid were added to a 100 ml flask, and refluxed for 3 h. After cooling to room temperature, the mixture was filtered. Colourless single crystals suitable for X-ray diffraction study were obtained by slow evaporation of a acetone-methanol (1:2, *v*/*v*) solution over a period of 2 d.

### Refinement

All H atoms were initially located in a difference Fourier map. C-atoms bound H atoms were constrained to ideal geometry with C—H = 0.93 Å for aryl, 0.97 Å for the methylene, and 0.96 Å for the methyl H atoms, while O—H = 0.82 Å and N—H = 0.86 Å were applied. H-atoms displacement values were constarined as *U*_iso_(H) = 1.2*U*_eq_(C,*N*), or 1.5*U*_eq_(C) for the methyl groups, and 1.5*U*_eq_(O).

### Crystal data

**Table:**

C_19_H_15_N_3_O_4_·CH_4_O	*F*(000) = 800
*M**_r_* = 381.38	*D*_x_ = 1.355 Mg m^−^^3^
Monoclinic, *P*2_1_/*c*	Mo *K*α radiation, λ = 0.71073 Å
Hall symbol: -P 2ybc	Cell parameters from 1033 reflections
*a* = 10.1166 (18) Å	θ = 2.9–20.4°
*b* = 11.095 (2) Å	µ = 0.10 mm^−^^1^
*c* = 18.510 (3) Å	*T* = 295 K
β = 115.896 (7)°	Block, colorless
*V* = 1869.0 (6) Å^3^	0.22 × 0.19 × 0.18 mm
*Z* = 4	

### Data collection

**Table:**

Bruker SMART CCD area-detector diffractometer	3302 independent reflections
Radiation source: fine-focus sealed tube	1666 reflections with *I* > 2σ(*I*)
graphite	*R*_int_ = 0.046
φ and ω scans	θ_max_ = 25.1°, θ_min_ = 2.2°
Absorption correction: multi-scan (*SADABS*; Sheldrick, 1996)	*h* = −9→12
*T*_min_ = 0.979, *T*_max_ = 0.982	*k* = −13→11
9663 measured reflections	*l* = −22→15

### Refinement

**Table:**

Refinement on *F*^2^	Primary atom site location: structure-invariant direct methods
Least-squares matrix: full	Secondary atom site location: difference Fourier map
*R*[*F*^2^ > 2σ(*F*^2^)] = 0.054	Hydrogen site location: inferred from neighbouring sites
*wR*(*F*^2^) = 0.161	H-atom parameters constrained
*S* = 1.02	*w* = 1/[σ^2^(*F*_o_^2^) + (0.0693*P*)^2^ + 0.0146*P*] where *P* = (*F*_o_^2^ + 2*F*_c_^2^)/3
3302 reflections	(Δ/σ)_max_ < 0.001
255 parameters	Δρ_max_ = 0.20 e Å^−^^3^
0 restraints	Δρ_min_ = −0.20 e Å^−^^3^

### Fractional atomic coordinates and isotropic or equivalent isotropic displacement parameters (Å^2^)

**Table:**

	*x*	*y*	*z*	*U*_iso_*/*U*_eq_	
O1	0.63801 (19)	0.10228 (18)	0.61156 (11)	0.0599 (6)	
O2	0.9689 (2)	−0.0368 (2)	0.74781 (14)	0.0785 (7)	
O3	1.7457 (2)	0.4174 (2)	0.71168 (15)	0.0813 (7)	
H3	1.8346	0.4274	0.7284	0.122*	
O4	1.8166 (2)	0.2717 (2)	0.80207 (16)	0.1010 (9)	
O5	0.7722 (3)	0.3037 (3)	0.55054 (17)	0.1139 (11)	
H5	0.6857	0.2843	0.5343	0.171*	
N1	0.4754 (3)	0.2645 (3)	0.50135 (15)	0.0710 (8)	
N2	0.9278 (2)	0.1277 (2)	0.66847 (14)	0.0584 (7)	
H2	0.8646	0.1762	0.6347	0.070*	
N3	1.0764 (2)	0.1439 (2)	0.69304 (14)	0.0571 (7)	
C1	0.3936 (4)	0.3439 (4)	0.4474 (2)	0.0946 (12)	
H1	0.4414	0.4064	0.4351	0.113*	
C2	0.2410 (4)	0.3411 (4)	0.4076 (2)	0.1093 (14)	
H2A	0.1892	0.3998	0.3700	0.131*	
C3	0.1702 (4)	0.2509 (4)	0.4249 (2)	0.0895 (12)	
H3A	0.0682	0.2465	0.3988	0.107*	
C4	0.2502 (3)	0.1634 (3)	0.48242 (18)	0.0657 (9)	
C5	0.1831 (4)	0.0687 (4)	0.5031 (2)	0.0820 (11)	
H5A	0.0813	0.0612	0.4782	0.098*	
C6	0.2653 (4)	−0.0124 (3)	0.5593 (2)	0.0830 (11)	
H6	0.2192	−0.0747	0.5732	0.100*	
C7	0.4194 (3)	−0.0038 (3)	0.5971 (2)	0.0694 (9)	
H7	0.4742	−0.0604	0.6356	0.083*	
C8	0.4887 (3)	0.0864 (3)	0.57778 (16)	0.0542 (8)	
C9	0.4050 (3)	0.1729 (3)	0.51962 (17)	0.0563 (8)	
C10	0.7231 (3)	0.0167 (3)	0.67118 (18)	0.0582 (8)	
H10A	0.6963	−0.0640	0.6495	0.070*	
H10B	0.7007	0.0238	0.7169	0.070*	
C11	0.8852 (3)	0.0348 (3)	0.69869 (19)	0.0569 (8)	
C12	1.1136 (3)	0.2365 (3)	0.66626 (17)	0.0616 (8)	
H12	1.0418	0.2893	0.6324	0.074*	
C13	1.2683 (3)	0.2610 (3)	0.68819 (17)	0.0553 (8)	
C14	1.3077 (3)	0.3561 (3)	0.65354 (18)	0.0634 (9)	
H14	1.2351	0.4054	0.6168	0.076*	
C15	1.4531 (3)	0.3789 (3)	0.67266 (17)	0.0611 (8)	
H15	1.4774	0.4426	0.6481	0.073*	
C16	1.5624 (3)	0.3087 (3)	0.72761 (17)	0.0529 (8)	
C17	1.5237 (3)	0.2143 (3)	0.76234 (19)	0.0661 (9)	
H17	1.5969	0.1660	0.7996	0.079*	
C18	1.3783 (3)	0.1893 (3)	0.74314 (18)	0.0634 (9)	
H18	1.3544	0.1246	0.7671	0.076*	
C19	1.7212 (3)	0.3295 (3)	0.7518 (2)	0.0640 (9)	
C20	0.7908 (5)	0.3609 (5)	0.4924 (3)	0.158 (2)	
H20A	0.8434	0.4347	0.5130	0.236*	
H20B	0.6967	0.3786	0.4491	0.236*	
H20C	0.8459	0.3107	0.4731	0.236*	

### Atomic displacement parameters (Å^2^)

**Table:**

	*U*^11^	*U*^22^	*U*^33^	*U*^12^	*U*^13^	*U*^23^
O1	0.0316 (11)	0.0766 (15)	0.0663 (12)	−0.0052 (9)	0.0166 (9)	0.0076 (11)
O2	0.0439 (13)	0.0783 (16)	0.1085 (17)	0.0127 (11)	0.0290 (12)	0.0269 (14)
O3	0.0437 (13)	0.0831 (18)	0.1124 (18)	−0.0145 (12)	0.0296 (13)	0.0009 (14)
O4	0.0376 (13)	0.126 (2)	0.120 (2)	0.0027 (14)	0.0169 (13)	0.0300 (18)
O5	0.0558 (16)	0.152 (3)	0.114 (2)	−0.0054 (17)	0.0187 (15)	0.065 (2)
N1	0.0464 (16)	0.096 (2)	0.0650 (16)	0.0006 (15)	0.0193 (13)	0.0115 (15)
N2	0.0301 (13)	0.0731 (18)	0.0675 (16)	0.0000 (11)	0.0173 (12)	0.0058 (13)
N3	0.0347 (14)	0.0673 (19)	0.0693 (16)	−0.0030 (12)	0.0226 (12)	−0.0025 (13)
C1	0.063 (3)	0.113 (3)	0.090 (3)	−0.002 (2)	0.017 (2)	0.034 (2)
C2	0.061 (3)	0.146 (4)	0.099 (3)	0.015 (3)	0.014 (2)	0.035 (3)
C3	0.042 (2)	0.130 (4)	0.079 (2)	0.001 (2)	0.0101 (19)	0.003 (2)
C4	0.0347 (17)	0.097 (3)	0.0587 (19)	−0.0039 (17)	0.0137 (15)	−0.0129 (18)
C5	0.0358 (19)	0.116 (3)	0.086 (2)	−0.015 (2)	0.0189 (18)	−0.018 (2)
C6	0.051 (2)	0.097 (3)	0.102 (3)	−0.026 (2)	0.034 (2)	−0.012 (2)
C7	0.0426 (18)	0.082 (2)	0.082 (2)	−0.0115 (17)	0.0262 (17)	0.0001 (18)
C8	0.0351 (16)	0.072 (2)	0.0551 (18)	−0.0051 (15)	0.0197 (14)	−0.0090 (15)
C9	0.0375 (17)	0.078 (2)	0.0539 (17)	−0.0065 (15)	0.0199 (14)	−0.0074 (16)
C10	0.0399 (17)	0.066 (2)	0.0674 (19)	−0.0008 (15)	0.0222 (15)	0.0061 (16)
C11	0.0386 (17)	0.063 (2)	0.071 (2)	−0.0008 (15)	0.0256 (16)	0.0017 (17)
C12	0.0340 (17)	0.074 (2)	0.069 (2)	0.0005 (15)	0.0151 (15)	0.0062 (17)
C13	0.0381 (17)	0.062 (2)	0.0602 (18)	−0.0018 (14)	0.0167 (14)	−0.0031 (15)
C14	0.0411 (18)	0.070 (2)	0.069 (2)	0.0000 (15)	0.0145 (15)	0.0123 (16)
C15	0.0451 (19)	0.063 (2)	0.070 (2)	−0.0090 (15)	0.0208 (16)	0.0031 (16)
C16	0.0358 (16)	0.057 (2)	0.0625 (18)	−0.0063 (14)	0.0180 (14)	−0.0115 (15)
C17	0.0411 (18)	0.073 (2)	0.078 (2)	0.0042 (16)	0.0203 (16)	0.0082 (18)
C18	0.0443 (18)	0.067 (2)	0.079 (2)	0.0015 (15)	0.0269 (16)	0.0113 (17)
C19	0.044 (2)	0.069 (2)	0.076 (2)	−0.0083 (17)	0.0242 (18)	−0.0094 (19)
C20	0.088 (4)	0.201 (6)	0.163 (5)	0.004 (3)	0.036 (3)	0.073 (4)

### Geometric parameters (Å, °)

**Table:**

O1—C8	1.371 (3)	C6—C7	1.406 (4)
O1—C10	1.425 (3)	C6—H6	0.9300
O2—C11	1.225 (3)	C7—C8	1.357 (4)
O3—C19	1.313 (4)	C7—H7	0.9300
O3—H3	0.8200	C8—C9	1.415 (4)
O4—C19	1.193 (4)	C10—C11	1.503 (4)
O5—C20	1.332 (5)	C10—H10A	0.9700
O5—H5	0.8200	C10—H10B	0.9700
N1—C1	1.318 (4)	C12—C13	1.461 (4)
N1—C9	1.365 (4)	C12—H12	0.9300
N2—C11	1.332 (3)	C13—C14	1.382 (4)
N2—N3	1.379 (3)	C13—C18	1.384 (4)
N2—H2	0.8600	C14—C15	1.378 (4)
N3—C12	1.267 (3)	C14—H14	0.9300
C1—C2	1.390 (5)	C15—C16	1.372 (4)
C1—H1	0.9300	C15—H15	0.9300
C2—C3	1.348 (5)	C16—C17	1.372 (4)
C2—H2A	0.9300	C16—C19	1.486 (4)
C3—C4	1.407 (5)	C17—C18	1.381 (4)
C3—H3A	0.9300	C17—H17	0.9300
C4—C5	1.393 (5)	C18—H18	0.9300
C4—C9	1.413 (4)	C20—H20A	0.9600
C5—C6	1.351 (5)	C20—H20B	0.9600
C5—H5A	0.9300	C20—H20C	0.9600
			
C8—O1—C10	116.1 (2)	C11—C10—H10A	109.2
C19—O3—H3	109.5	O1—C10—H10B	109.2
C20—O5—H5	109.5	C11—C10—H10B	109.2
C1—N1—C9	117.5 (3)	H10A—C10—H10B	107.9
C11—N2—N3	118.0 (2)	O2—C11—N2	124.6 (3)
C11—N2—H2	121.0	O2—C11—C10	117.6 (3)
N3—N2—H2	121.0	N2—C11—C10	117.8 (3)
C12—N3—N2	116.3 (2)	N3—C12—C13	120.5 (3)
N1—C1—C2	124.9 (4)	N3—C12—H12	119.8
N1—C1—H1	117.6	C13—C12—H12	119.8
C2—C1—H1	117.6	C14—C13—C18	118.5 (3)
C3—C2—C1	118.2 (4)	C14—C13—C12	120.3 (3)
C3—C2—H2A	120.9	C18—C13—C12	121.2 (3)
C1—C2—H2A	120.9	C15—C14—C13	120.9 (3)
C2—C3—C4	120.2 (3)	C15—C14—H14	119.6
C2—C3—H3A	119.9	C13—C14—H14	119.6
C4—C3—H3A	119.9	C16—C15—C14	120.7 (3)
C5—C4—C3	122.7 (3)	C16—C15—H15	119.7
C5—C4—C9	119.5 (3)	C14—C15—H15	119.7
C3—C4—C9	117.8 (3)	C15—C16—C17	118.6 (3)
C6—C5—C4	120.3 (3)	C15—C16—C19	123.4 (3)
C6—C5—H5A	119.9	C17—C16—C19	118.0 (3)
C4—C5—H5A	119.9	C16—C17—C18	121.4 (3)
C5—C6—C7	120.9 (3)	C16—C17—H17	119.3
C5—C6—H6	119.5	C18—C17—H17	119.3
C7—C6—H6	119.5	C17—C18—C13	119.9 (3)
C8—C7—C6	120.5 (3)	C17—C18—H18	120.1
C8—C7—H7	119.8	C13—C18—H18	120.1
C6—C7—H7	119.8	O4—C19—O3	123.5 (3)
C7—C8—O1	124.6 (3)	O4—C19—C16	123.4 (3)
C7—C8—C9	119.7 (3)	O3—C19—C16	113.1 (3)
O1—C8—C9	115.7 (3)	O5—C20—H20A	109.5
N1—C9—C4	121.5 (3)	O5—C20—H20B	109.5
N1—C9—C8	119.3 (3)	H20A—C20—H20B	109.5
C4—C9—C8	119.2 (3)	O5—C20—H20C	109.5
O1—C10—C11	112.0 (2)	H20A—C20—H20C	109.5
O1—C10—H10A	109.2	H20B—C20—H20C	109.5
			
C11—N2—N3—C12	−176.6 (3)	O1—C8—C9—C4	−179.5 (2)
C9—N1—C1—C2	−0.2 (6)	C8—O1—C10—C11	174.5 (2)
N1—C1—C2—C3	0.0 (7)	N3—N2—C11—O2	1.1 (5)
C1—C2—C3—C4	0.5 (6)	N3—N2—C11—C10	−179.4 (2)
C2—C3—C4—C5	179.6 (4)	O1—C10—C11—O2	−177.3 (3)
C2—C3—C4—C9	−0.9 (6)	O1—C10—C11—N2	3.1 (4)
C3—C4—C5—C6	−179.5 (3)	N2—N3—C12—C13	−179.4 (2)
C9—C4—C5—C6	1.1 (5)	N3—C12—C13—C14	174.4 (3)
C4—C5—C6—C7	−0.8 (5)	N3—C12—C13—C18	−4.9 (5)
C5—C6—C7—C8	0.0 (5)	C18—C13—C14—C15	0.5 (5)
C6—C7—C8—O1	179.7 (3)	C12—C13—C14—C15	−178.9 (3)
C6—C7—C8—C9	0.5 (5)	C13—C14—C15—C16	−1.0 (5)
C10—O1—C8—C7	−0.3 (4)	C14—C15—C16—C17	0.8 (4)
C10—O1—C8—C9	179.0 (2)	C14—C15—C16—C19	−179.0 (3)
C1—N1—C9—C4	−0.1 (5)	C15—C16—C17—C18	−0.1 (5)
C1—N1—C9—C8	−179.4 (3)	C19—C16—C17—C18	179.8 (3)
C5—C4—C9—N1	−179.8 (3)	C16—C17—C18—C13	−0.5 (5)
C3—C4—C9—N1	0.7 (5)	C14—C13—C18—C17	0.3 (5)
C5—C4—C9—C8	−0.5 (4)	C12—C13—C18—C17	179.6 (3)
C3—C4—C9—C8	180.0 (3)	C15—C16—C19—O4	176.7 (3)
C7—C8—C9—N1	179.1 (3)	C17—C16—C19—O4	−3.2 (5)
O1—C8—C9—N1	−0.3 (4)	C15—C16—C19—O3	−3.9 (4)
C7—C8—C9—C4	−0.2 (4)	C17—C16—C19—O3	176.3 (3)

### Hydrogen-bond geometry (Å, °)

**Table:**

*D*—H···*A*	*D*—H	H···*A*	*D*···*A*	*D*—H···*A*
O3—H3···O2^i^	0.82	1.88	2.695 (3)	171
O5—H5···N1	0.82	1.95	2.765 (3)	171
N2—H2···O5	0.86	2.01	2.838 (4)	162

Symmetry codes: (i) −*x*+3, *y*+1/2, −*z*+3/2.
